# Correction: A β-Lactamase Based Reporter System for ESX Dependent Protein Translocation in Mycobacteria

**DOI:** 10.1371/annotation/f03f7456-0e04-4c4b-a606-51f262900e8d

**Published:** 2013-01-07

**Authors:** Tobias Rosenberger, Juliane K. Brülle, Peter Sander

The authors request the retraction of the publication “A β-lactamase based reporter system for ESX dependent protein translocation in mycobacteria” because the main conclusions of the publication are unreliable. The main finding from the publication was the development of an ESX (precisely ESX-3) dependent protein translocation reporter based on an ampicillin resistant phenotype. Subsequent investigations by the authors have demonstrated that the reporter construct “pIV-blaTEM”, which was supposed to express the “Bla-TEM-EsxG” fusion protein, was accidentally replaced with a vector expressing a fusion protein carrying a twin-arginine-transporter (tat) secretion signal (not referenced in the publication). Re-testing of a newly constructed “true” BlaTEM-EsxG reporter strain indicated that the vector pIV-blaTEM does not confer an ampicillin-resistance phenotype. The second ESX reporter construct (pVI-blaTEM) expressing BlaTEM-EsxB does confer an ampicillin-resistance phenotype, but this phenotype does not depend on a functional ESX-1 secretion system as demonstrated by characterization of a subsequently generated eccD1 (MSMEG_0068) deletion mutant expressing BlaTEM-EsxB.

